# Deciphering transcriptomic signatures in schizophrenia, bipolar disorder, and major depressive disorder

**DOI:** 10.3389/fpsyt.2025.1574458

**Published:** 2025-07-14

**Authors:** Priyanka, Rajesh Kumar, Vinod Kumar, Ashwani Kumar, Sandeep Singh Rana

**Affiliations:** ^1^ Department of Applied Psychology, Guru Jambheshwar University of Science & Technology, Hisar, India; ^2^ CSIR - Bioinformatics Centre, Institute of Microbial Technology, Chandigarh, India; ^3^ Skill Vishawkarma University, Palwal, India

**Keywords:** bipolar disorder, major depressive disorder, schizophrenia, transcriptomics, mental health

## Abstract

Schizophrenia (SCZ), Bipolar Disorder (BD), and Major Depressive Disorder (MDD) are severe psychiatric conditions that share overlapping clinical symptoms, yet they differ in their underlying molecular mechanisms. Despite extensive research, the biological foundations of these disorders remain incompletely understood. In this study, we performed a large-scale transcriptomic analysis by integrating 557 publicly available RNA-seq datasets from post-mortem brain tissues, spanning multiple regions, to better understand the shared and distinct molecular features of these disorders. Using systematic bioinformatic approaches, we identified differentially expressed genes (DEGs) and investigated associated biological pathways, regulatory transcription factors, and drug-gene interactions. Our analysis revealed notable overlap in gene expression profiles, particularly between SCZ and BD, suggesting common molecular pathways underlying these disorders. At the same time, each disorder also demonstrated unique transcriptional patterns, supporting the existence of disorder-specific mechanisms. Brain region-specific analyses further highlighted spatial heterogeneity in gene expression, with significant differences observed in regions such as the hippocampus and dorsolateral prefrontal cortex (DLPFC). The transcription factor enrichment analysis revealed distinct regulatory programs driving each disorder: MDD pathology appears regulated by ASCL3, MYOG, HNF1B, RUNX3, FOXA1 and STAT4; BD exhibited predominant control by immune-regulatory factors including FOSL1, FOSL2, PLSCR1, RELB, BATF3, IRF and NFKB1; while SCZ demonstrated unique regulation through ATF5, CREB3L3, SNAI1, NFIL3, CEBPB, RELB and IRF transcription factors. Moreover, our drug-gene interaction analysis uncovered promising therapeutic targets, with several differentially expressed genes showing potential for drug repurposing, particularly in relation to antipsychotics and immunomodulatory agents. Our comprehensive transcriptomic analysis reveals both shared molecular mechanisms and distinct immune signatures across schizophrenia, bipolar disorder, and major depressive disorder, advancing our understanding of psychiatric pathophysiology while highlighting the heterogeneous nature of these conditions. These findings establish a critical foundation for developing targeted, patient-specific therapeutic interventions that address the underlying biological complexity of major psychiatric disorders.

## Introduction

Psychiatric disorders represent a significant global health burden, affecting millions worldwide and posing substantial challenges to healthcare systems, social structures, and economic frameworks (1). Among these, Schizophrenia (SCZ), Bipolar Disorder (BD), and Major Depressive Disorder (MDD) stand as particularly impactful conditions, characterized by complex symptomatology and often devastating effects on individual functioning and quality of life (2). Despite their clinical significance, the underlying molecular mechanisms driving these disorders remain incompletely understood, hindering the development of more effective therapeutic strategies. The global burden of these disorders is substantial, with approximately 1% of the population affected by SCZ, 2.4% by BD, and 3.8% by MDD (1, 3). These conditions account for a significant proportion of disability-adjusted life years (DALYs) worldwide.

The overlapping symptomatology and high comorbidity rates among these disorders suggest shared biological underpinnings, yet each condition also exhibits unique clinical features that point to distinct pathophysiological mechanisms (4). Recent advances in high-throughput sequencing technologies and bioinformatics have provided unprecedented opportunities to explore these biological foundations at the molecular level (5). Transcriptomic analysis, in particular, has emerged as a powerful tool for understanding the complex interplay of genetic and environmental factors in psychiatric disorders (5).

The complexity of psychiatric disorders is further compounded by the involvement of multiple brain regions and neural circuits. The dorsolateral prefrontal cortex (DLPFC) and hippocampus have been consistently implicated in the pathophysiology of SCZ, BD, and MDD, yet their relative contributions to each disorder remain debated (6). Understanding region-specific transcriptional changes is crucial for developing targeted therapeutic approaches and identifying biomarkers for early diagnosis and intervention.

Previous studies have typically focused on individual disorders or specific brain regions, limiting our understanding of the broader biological landscape across psychiatric conditions (5, 7–9). The integration of data from multiple studies and brain regions offers a unique opportunity to identify both shared and unique molecular signatures, potentially revealing novel therapeutic targets and biological pathways (10). This comprehensive approach is particularly relevant given the growing recognition of psychiatric disorders as existing along a biological continuum rather than as discrete entities.

Recent technological advances have enabled more sophisticated analyses of gene expression patterns and pathway dysregulation, providing new insights into the molecular basis of psychiatric disorders (5). The application of advanced bioinformatic approaches to large-scale transcriptomic data has revealed complex patterns of gene expression changes and pathway perturbations that may underlie the development and progression of these conditions. The integration of machine learning approaches with transcriptomic analysis has further enhanced our ability to identify complex patterns and relationships within large-scale genomic data (11). These computational advances have enabled more robust identification of disease-specific signatures and pathway interactions.

In this study, we analyze transcriptomic data from SCZ, BD, and MDD across multiple brain regions to address the specific questions of - (i) identifying shared and unique genomic signatures across disorders, (ii) characterizing brain region-specific transcriptional patterns, (iii) elucidating distinct molecular mechanisms through pathway analysis, and (iv) exploring the implications of these findings for therapeutic development.

## Methodology

### Dataset collection

We selected and searched the publicly available dataset on BD, SCZ, and MDD, with considerations of the Preferred reporting items for systematic reviews and meta-analysis (PRISMA) guidelines, ensuring a systematic and thorough review of the available data (12). The selection criteria include - i) Study should involve human subjects (“Homo sapiens”), ii) Study should contain a minimum of 12 samples to ensure statistical robustness in downstream analysis, iii) data should be publicly available. The following exclusion criteria was used - i) Study should include the strict patient vs control analysis, ii) Should contain experimental samples other than post-mortem brain samples, iii) experimental data excluding cellular populations. Following these data guidelines and criteria, we have been left with 5 RNA-Sequencing datasets - GSE138082, GSE174407, GSE80655, GSE78936, and GSE379666. However, for the GSE174407 study, the raw sequence read files were not publicly accessible and consequently, we had to exclude this dataset from our further analysis.

## Transcriptomic dataset processing

We downloaded the raw FASTQ files for the selected datasets using the NCBI SRA Toolkit via a custom Bash script, which is now provided as a **Supplementary File**. The sequencing data were processed using the nf-core/rnaseq pipeline (version 3.14.0) (https://nf-co.re/rnaseq/3.14.0/), with default parameters. This pipeline incorporates a comprehensive suite of tools for quality control, read preprocessing, alignment, and quantification. Initial processing steps included quality assessment using FastQC and adapter trimming with Trim Galore, which removes low-quality bases (Phred score < 20) and sequencing adapters. Trimmed reads were aligned to the human reference genome (GRCh38, release 113) using STAR, and transcript quantification was performed using Salmon at both gene and transcript levels. To further evaluate data quality, we utilized additional metrics and tools integrated in the pipeline: Qualimap for gene body coverage analysis, RSeQC for read distribution assessment, Picard Tools for alignment quality statistics, and TIN score computation to assess RNA integrity. Normalized bigWig files were also generated for downstream visualization. While the pipeline does not automatically filter out low-quality samples, we carefully reviewed all quality control reports (aggregated using MultiQC) and excluded any samples that failed to meet acceptable quality standards based on metrics such as low mapping percentage, poor read quality, or abnormal coverage profiles. All analysis steps were executed using nextflow, enabling reproducibility, traceability, and scalable execution across datasets and computing environments.

## Batch correction and visualization

The limma R package (version 3.20) was used to remove any potential batch effects in the dataset. The batch effect correction for each study was done by using the known variable such as sequencing library preparations (13). Unknown batch effects were further estimated using the checking of the surrogate variables by the limma R package. For plotting and visualization of the batch effect, the first two principal components are considered. Further downstream analysis has been done with a batch-corrected data matrix which consists of 19447 human protein-coding genes. To represent the latent projection of all the samples in a 2-dimensional space we have also performed the Uniform manifold approximation projection (UMAP) projection on a batch corrected data matrix. UMAP analysis was performed on each tissue and the disease class which the sample belongs to. In addition, we also performed the UMAP analysis for each distinct brain region, examining samples across disease conditions.

## Differential expression analysis and identification of key genes and pathways

To investigate transcriptional changes across different brain regions and disease conditions, we performed comprehensive differential expression analysis using DESeq2 (14). This robust analytical approach enabled us to identify genes that showed significant alterations in expression patterns when compared to control samples. For statistical stringency and biological relevance, we established dual filtering criteria: an absolute log2 fold change threshold exceeding 1 (corresponding to a minimum two-fold change in expression) and a statistical significance threshold of padj < 0.05. This balanced approach helped us identify genes that showed both substantial magnitude of change and statistical reliability. The differentially expressed genes were then examined for their functional implications through Gene Set Enrichment Analysis (GSEA) (15). We incorporated pathway information from two complementary databases: the Reactome database, which provides detailed molecular pathway annotations, and the Molecular Signatures Database (MSigDB), offering a broader spectrum of functional gene sets (15). This dual-database approach enabled us to capture both specific molecular mechanisms and broader biological processes affected in each disease condition. By analyzing each disease group separately, we could identify both unique pathway perturbations specific to individual conditions and common pathway alterations shared across multiple disease states.

## Transcription factor enrichment

To elucidate the regulatory mechanisms underlying the identified pathways, we performed transcription factor (TF) enrichment analysis using ChIP-X Enrichment Analysis 3 (ChEA3), a database which integrated experimental evidence of the identified TF (12). This analysis was crucial for understanding the upstream regulators that orchestrate the observed pathway-specific gene expression patterns. By analyzing the leading genes derived from MSigDB and Reactome pathway analyses, we sought to identify both unique and shared transcriptional regulators across different pathways. ChEA3’s integrative approach, which combines multiple lines of evidence including RNA-seq-based TF-gene co-expression data, ChIP-seq-derived TF-target associations, and TF-gene co-occurrence patterns from crowd-sourced gene lists, provided a comprehensive view of the regulatory landscape. The composite ranking system of ChEA3 enhanced the reliability of our TF predictions by synthesizing evidence from these diverse data sources, offering insights into the hierarchical organization of the transcriptional networks governing these pathways. This approach enabled us to identify key regulatory nodes that could explain the observed pathwayspecific gene expression patterns and potential crosstalk between different biological processes.

## Identification of key drugs for therapeutics

We have utilized the Drug-gene interaction database (DGIdb) resource (16) (https://www.dgidb.org/search_interactions) for the purpose of studying the therapeutic potentials of the identified leading genes governing each pathway and thus sheds light upon a unique treatment-targeted approach. This database maintains the latest information on drug-gene interactions identified from experimental studies. We have searched our signature genes in the database and identified their association, in order to search for disease specific therapeutic interventions.

## Results

### Dataset characteristics and study overview

Brain regions including the cortex, amygdala, and hippocampus were selected based on extensive prior evidence highlighting their key roles in cognition, emotional regulation, and memory, all of which are critically affected in major psychiatric disorders. Following PRISMA guidelines (17), we left with four studies, to be included in our analysis. The study PRJNA314463 (GSE78936) consists of samples from 24 controls, 30 BD and 28 SCZ, whereas Study PRJNA319583 (GSE80655) have 87 control, 87 BD, 94 MDD, and 83 SCZ patients, study PRJNA379666 (GSE379666) consisted of 24 control and 22 SCZ samples, and study PRJNA574470 (GSE138082) was made up of 39 control and 39 SCZ (**Supplementary Table S1**; **Table 1**). The complete dataset was accessed and process in October 2023. The complete description of the dataset used in the present study has been added to **Supplementary Table S2**.

**Table 1:** Summary of the dataset used in the present study.

ID	Disease	Brain areas	Platform	File type	Reference
GSE78936	Bipolar and Schizophrenia	Orbitofrontal Cortex	Illumina HiSeq 2000 (Homo sapiens)	Raw Fastq	(17)
GSE80655	Bipolar, Schizophrenia and Major Depressive Disorder	Anterior Cingulate Cortex, Dorsolateral Prefrontal Cortex	Illumina HiSeq 2000 (Homo sapiens)	Raw Fastq	(18)
GSE379666	Schizophrenia	Amygdala	Illumina HiSeq 2000 (Homo sapiens)	Raw Fastq	(19)
GSE138082	Schizophrenia	Hippocampus	Illumina HiSeq 2000 (Homo sapiens)	Raw Fastq	(20)

**Figure 1:**
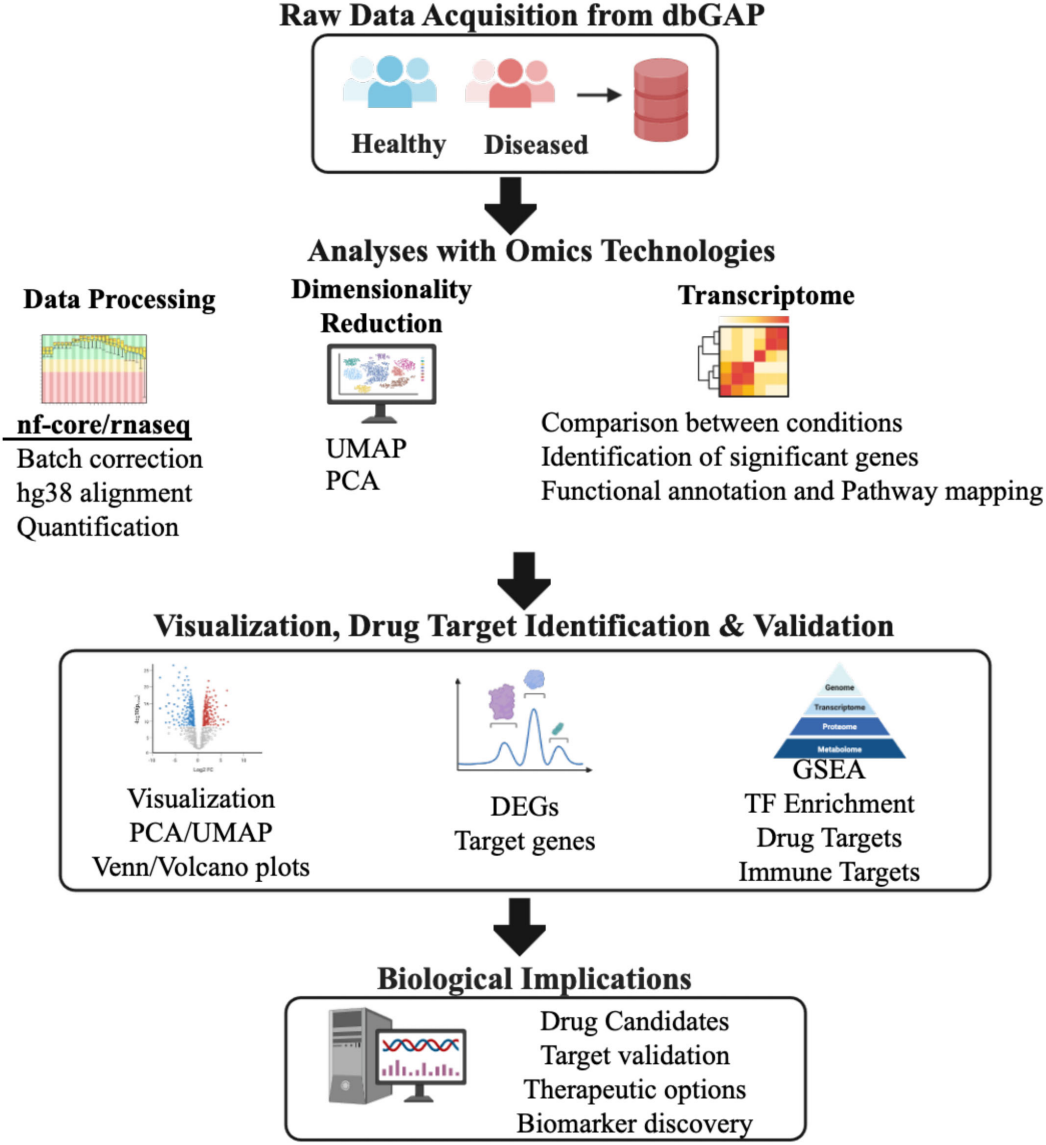
Schematic representation of the overall integrative bioinformatics pipeline used in the study. Computational pipeline overview for analyzing RNA-seq data from healthy and diseased samples (N = 558, **Supplementary Table S1**). Raw transcriptomic data from the Database of Genotypes and Phenotypes (dbGAP) underwent quality control and preprocessing using nf-core/rnaseq pipeline with human genome reference hg38 alignment. Data processing steps included batch effect correction, gene expression quantification, and normalization. Dimensionality reduction was performed using Principal Component Analysis (PCA) and Uniform Manifold Approximation and Projection (UMAP), where UMAP1 and UMAP2 represent the two primary embedding dimensions capturing non-linear data structure. Differential gene expression analysis identified significantly dysregulated genes (DEGs) using DESeq2 Wald test with statistical thresholds: log_2_fold-change ≥ 1.0 and padj-value < 0.05. Functional enrichment analysis employed Gene Set Enrichment Analysis (GSEA) and transcription factor (TF) enrichment to identify perturbed biological pathways and regulatory networks. Drug target identification and validation utilized the Drug-Gene Interaction database (DGIdb) to identify therapeutic candidates and immune-related targets for potential clinical translation. PCA, Principal Component Analysis; UMAP, Uniform Manifold Approximation Projection; DEG, Differentially Expressed Genes; GSEA, Gene Set Enrichment Analysis; TF, Transcription Factor.

Collectively, the present study consists of a cohort of 557 public RNA-seq dataset of BD, SCZ and MDD from different regions of the brain and accessed their genomic expression profile for the identification of unique and shared features. All samples were processed using standardized RNAseq protocols on the Illumina HiSeq 2000 platform, ensuring technical consistency across studies. The brain regions analyzed represent key areas implicated in psychiatric disorders: the orbitofrontal cortex, involved in decision-making and emotional processing; the anterior cingulate and dorsolateral prefrontal cortex, crucial for executive function and emotional regulation; the amygdala, central to fear and emotional responses; and the hippocampus, essential for memory formation and emotional processing. This diverse regional sampling allows for a comprehensive analysis of disease-specific molecular signatures across different functional brain areas. **Figure 1** provides the complete architecture of the study performed.

## Dimensionality reduction uncovers disease specific features across psychological conditions

**Figure 2:**
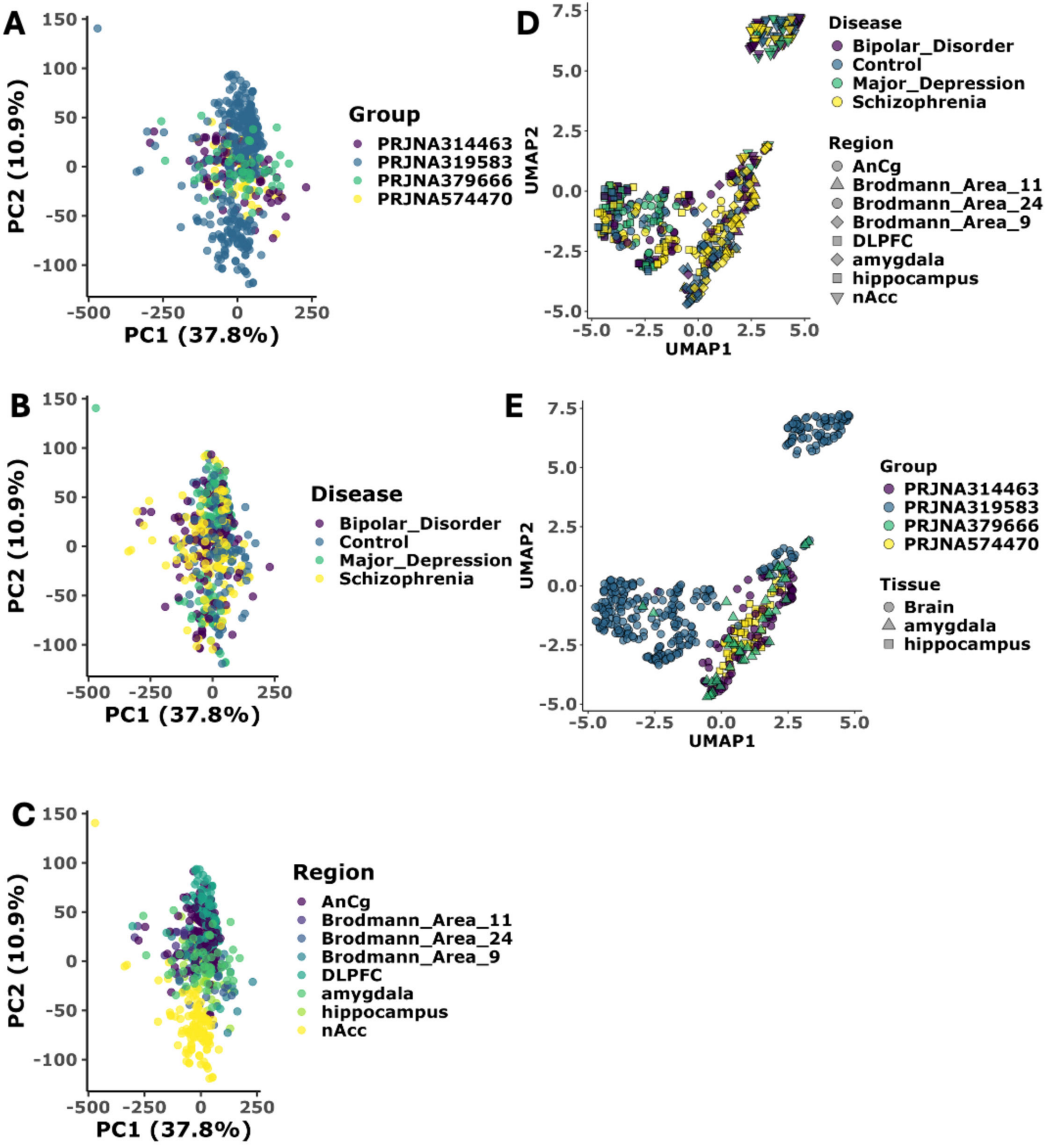
Dimensionality reduction validates disease specific pattern across conditions. **(A)** Principal Component Analysis (PCA) of transcriptomic data stratified by study type. PC1 and PC2 capture 37.8% and 10.9% of total variance, respectively (N = 557 samples, **Supplementary Table S1**). Sample distribution by study cohort (PRJNA314463 (N = 83), PRJNA319583 (N = 352), PRJNA379666 (N = 46), PRJNA574470 (N = 78)) demonstrates effective batch correction with no study-specific clustering (PERMANOVA p > 0.05). Each dot represents a patient sample colored by study cohort. All the sample cluster together showing batch correction has been successful. **(B)** Principal Component Analysis (PCA) of transcriptomic data stratified by disease category. Disease classification shows molecular convergence between neuropsychiatric disorders, indicating successful batch effect removal: Bipolar Disorder (BD), Major Depression (MDD), Schizophrenia (SCZ), and healthy controls. Each dot represents a patient sample colored by Disease. All the sample cluster together showing batch correction has been successful. **(C)** Principal Component Analysis (PCA) of transcriptomic data stratified by site of biopsy in brain. Brain region-specific analysis across eight anatomical regions: Anterior Cingulate Cortex (AnCg), Brodmann Areas 11, 24, and 9, Dorsolateral Prefrontal Cortex (DLPFC), amygdala, hippocampus, and Nucleus Accumbens (nAcc). Each dot represents a patient sample colored by brain region. All the sample cluster together showing batch correction has been successful. **(D)** UMAP projection of transcriptomic stratified by disease category and Site of biopsy UMAP1 and UMAP2 represent primary embedding dimensions preserving local and global data structure. UMAP projection colored by disease type shows clear clustering of samples by pathological condition, indicating successful removal of technical batch effects while preserving biological signal. Each point represents an individual sample, with point size corresponding to brain region type. **(E)** UMAP projection of transcriptomic stratified by study type and tissue type. Brain tissue region-stratified analysis confirms successful integration of samples across different brain anatomical regions, with point shapes representing regional identity and colors indicating disease status. Statistical significance assessed using Wilcoxon rank-sum test with Benjamini-Hochberg correction (adjusted p < 0.05).

The raw FASTQ files were processed using a uniform bioinformatics pipeline to minimize technical variability in the analysis workflow. The raw count matrix of 557 cases and control dataset were used for the batch correction to mitigate technical variations across the datasets. Before batch correction, distinct clustering was observed across study groups and conditions, reflecting potential batch effects. However, after batch correction, the samples from all groups intermingled, indicating the removal of these batch-specific variations (**Supplementary Figure 1**). Following batch correction, the Principal Component Analysis (PCA) revealed distinct patterns across psychiatric conditions, brain regions, and study groups. The study group specific PCA clustering, validates our batch correction approach, as samples from all studies (PRJNA314463, PRJNA319583, PRJNA379666, and PRJNA574470) showed uniform distribution without distinct study-specific clustering, confirming successful removal of batch effects (**Figure 2A**, top). Since the dataset consists of different psychological conditions and across several brain regions, we extend the PCA analysis across conditions and different brain regions.

PCA clustering based on the disease status (**Figure 2A**, middle), we observed partial overlapping between psychiatric conditions, with SCZ and BD samples showing closer molecular signatures compared to MDD, suggesting a molecular continuum among these disorders. Control samples showed a more diffuse distribution, indicating natural biological variability in healthy brain tissue (21). The brain region-specific analysis (bottom panel A) revealed interesting biological clustering, particularly in the hippocampus and DLPFC regions, which formed more distinct clusters compared to other brain areas, suggesting strong region-specific transcriptional signatures (**Figure 2A**, bottom). However, the limited variance explained by the first two principal components (PC1: 27.6%, PC2: 10.9%) indicated additional complexity in the data structure not captured by linear dimensional technique. To further uncover the more biological variations in the dataset, we extended our analysis with non-linear dimensionality reduction techniques. UMAP analysis reveals more nuanced biological patterns, particularly in disease-specific manner (**Figure 2B**). The UMAP projection shows that while there is partial overlap between SCZ and BD, MDD forms a distinct cluster with partial or no overlap with SCZ. These complementary analyses suggest that while these psychiatric disorders share common molecular features, they also possess unique transcriptional programs particularly for major depressive disorder, as evidenced by both PCA and UMAP.

## Shared and unique genomic signatures across psychiatric disorders

**Figure 3:**
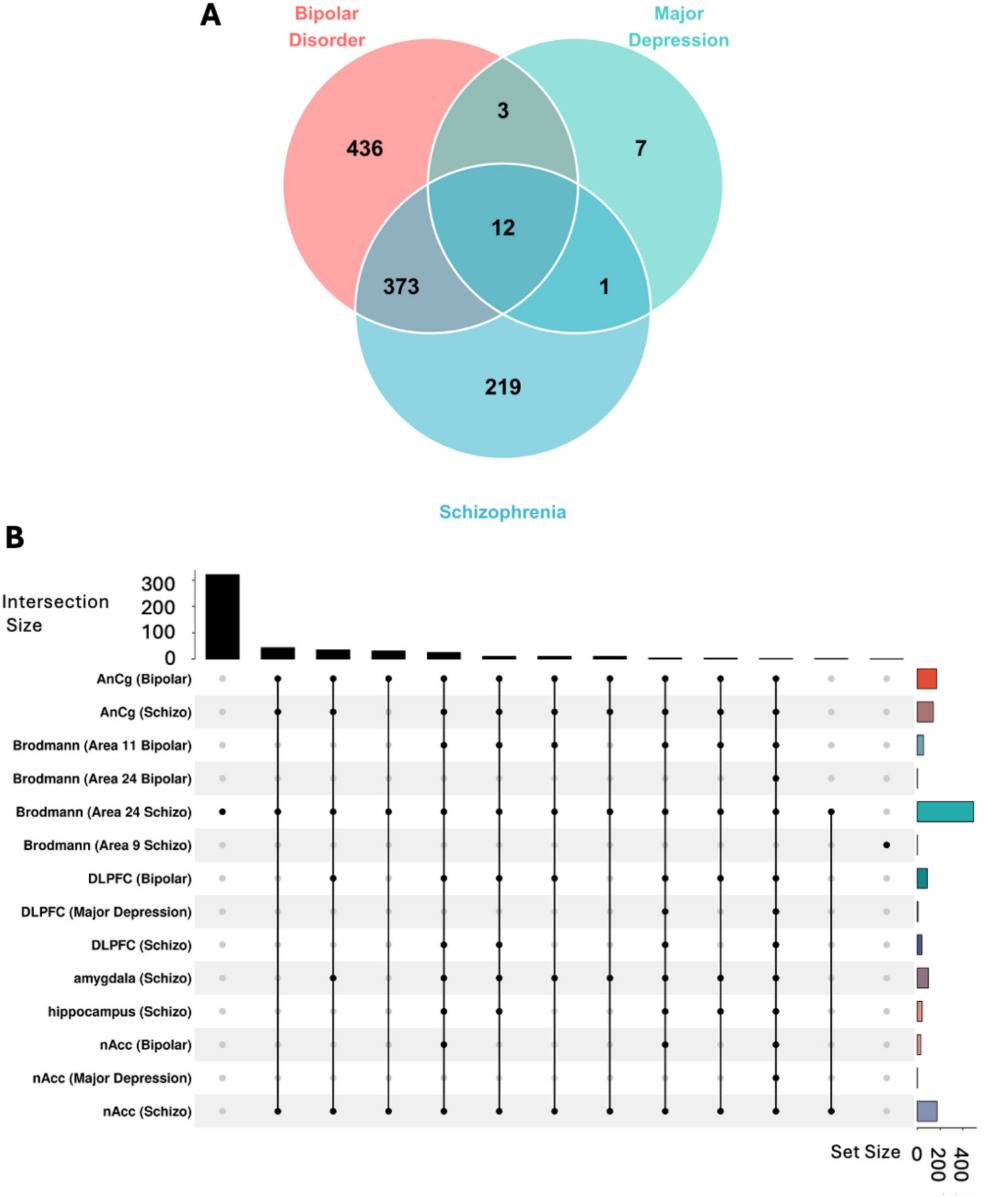
Transcriptomic analysis identify shared and unique genomic vulnerability across psychosis disorders. **(A)** Venn diagram analysis of differentially expressed genes (DEGs) across psychiatric conditions. Post-mortem brain tissue transcriptome analysis comparing Bipolar Disorder (BD) (N=117), Major Depression (MDD) (N=94), and Schizophrenia (SCZ) (N=173) versus healthy controls (N = 174). Differentially expressed genes (DGE) identified using DESeq2 with statistical thresholds: log_2_fold-change ≥ 1.0 and pdj-value < 0.05. Disease-specific DEGs: BD (436 unique genes), MDD (7 unique genes), SCZ (219 unique genes). Shared molecular signatures: 12 hub genes dysregulated across all three conditions, 373 genes shared between BD and SCZ, 3 genes shared between BD and MDD, and 1 gene shared between MDD and SCZ, indicating common pathobiological mechanisms underlying psychotic spectrum disorders. **(B)** UpSet plot visualization of brain region-specific transcriptomic patterns. Intersection analysis across eight brain regions: Anterior Cingulate Cortex (AnCg), Brodmann Areas 11, 24, and 9, Dorsolateral Prefrontal Cortex (DLPFC), amygdala, hippocampus, and Nucleus Accumbens (nAcc). Horizontal bars indicate total DEGs per region-disease combination; vertical bars show intersection sizes with connected dots representing shared gene sets. Statistical significance of overlaps assessed using hypergeometric distribution test (p < 0.05).

Our transcriptomic analysis revealed both unique and overlapping genomic signatures across psychiatric disorders (**Supplementary Table S3**). BD exhibited the largest number of unique DEG’s, followed by SCZ and MDD. Notably, BD and SCZ shared substantial molecular overlap with 373 common DEGs, suggesting significant biological convergence between these conditions (**Figure 3A**). We also identified 12 common hub genes across all the three disorders. The brain region-specific analysis demonstrated distinct patterns of transcriptional dysregulation. The amygdala and hippocampus demonstrated unique transcriptional signatures, especially in SCZ, highlighting the region-specific nature of psychiatric pathology (**Figure 3B**). This comprehensive analysis suggests a complex interplay between shared and unique genomic vulnerabilities across psychiatric disorders, with substantial overlap between BD and SCZ, while MDD shows more distinct molecular patterns.

### Pathway analysis reveals distinct molecular mechanisms in psychiatric disorders

**Figure 4:**
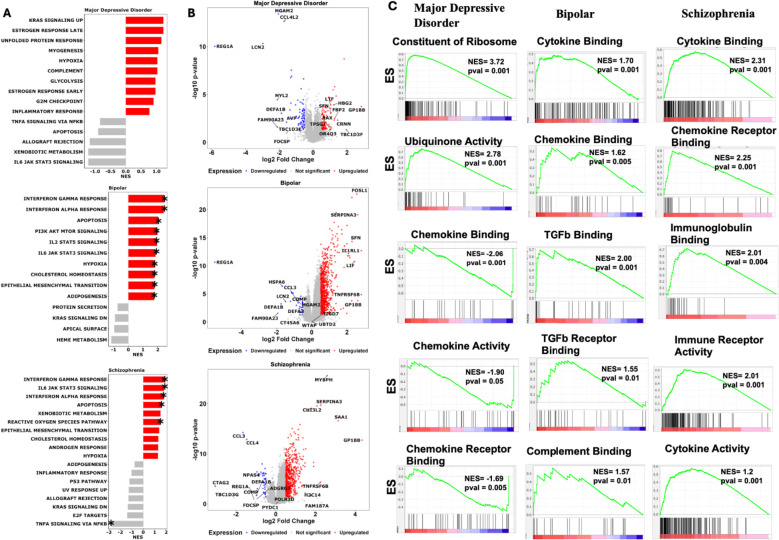
Pathway analysis uncovers the shared and unique biological pathway across psychosis disorders. **(A)** Hallmark pathway enrichment analysis across psychiatric disorders. Gene Set Enrichment Analysis (GSEA) comparing Major Depressive Disorder, Bipolar Disorder, and Schizophrenia against neurotypical controls using MSigDB Hallmark gene sets. Analysis performed with 1000 gene set permutations and DESeq2 normalized count. Red bars indicate significantly upregulated pathways (NES > 0, p < 0.05, FDR < 0.25), gray bars represent nonsignificant enrichment. Asterisks (*) denote statistical significance after Benjamini-Hochberg multiple testing correction. **(B)** Differential gene expression profiles in psychiatric disorders. Volcano plots displaying log_2_ fold change (x-axis) versus -log_10_ p-value (y-axis) for differentially expressed genes in each disorder compared to controls. Red dots represent significantly upregulated genes (log_2_FC > 0.5, p < 0.05), blue dots represent significantly downregulated genes (log_2_FC < -0.5, p < 0.05), and gray dots represent non-significant genes. Statistical analysis performed using DESeq2 with Wald test and Benjamini-Hochberg correction. Gene symbols for the most significantly altered genes are labeled for biological interpretation. **(C)** Gene Ontology enrichment analysis of immune system pathways. Pre-ranked GSEA enrichment plots for immune-related Gene Ontology Biological Process terms across psychiatric disorders. Green line shows running enrichment score along ranked gene list, with peak representing final Normalized Enrichment Score (NES). Vertical black lines indicate positions of genes within each immune gene set. Middle barcode plot displays gene rankings with red-to-blue gradient representing expression correlation with phenotype. Positive NES indicates pathway upregulation; negative NES indicates downregulation. Statistical significance determined by permutation testing (1000 permutations, p < 0.05, FDR < 0.25). Only top 5 pathway based on highest NES score and p-value are shown. GSEA, Gene Set Enrichment Analysis; NES, Normalized Enrichment Score; DEG, Differentially Expressed Gene; FC, Fold Change; FDR, False Discovery Rate; GO, Gene Ontology.

The pathway enrichment analysis uncovered distinct biological mechanisms underlying each psychiatric disorder. MDD showed predominant dysregulation in stress response and metabolic pathways, with significant enrichment in KRAS signaling, unfolded protein response, and glycolysis, suggesting cellular stress as a key pathogenic mechanism. BD demonstrated the most robust immune system activation, characterized by strong enrichment in interferon response pathways and JAK-STAT signaling cascades, alongside significant involvement of the PI3K-AKTmTOR pathway, indicating a complex interplay between immune regulation and cellular growth signaling. SCZ exhibited a unique combination of immune dysregulation, oxidative stress, and metabolic perturbations, with notable enrichment in interferon responses and reactive oxygen species pathways (**Figure 4**). Thus, the pathway enrichment analysis distinct molecular signatures with both convergent and divergent patterns across psychiatric disorders. While BD and SCZ showed striking similarities in immune system activation, with significant upregulation of interferon gamma and alpha response pathways, suggesting shared inflammatory mechanisms in their pathophysiology. In contrast, MDD exhibited a distinct pattern with downregulation of inflammatory responses and TNFα signaling, indicating that while immune system dysregulation is common across these disorders, the directional changes are disorder specific. Similar to the immune system the metabolic pathways show disease specific patterns. MDD demonstrated upregulation of fundamental metabolic processes including glycolysis, hypoxia response, and estrogen signaling, suggesting cellular stress and altered energy metabolism as key features. BD uniquely showed strong activation of PI3K-AKT-mTOR and JAK-STAT signaling cascades, alongside upregulated cholesterol homeostasis and epithelial-mesenchymal transition pathways, indicating disrupted cellular signaling and plasticity. SCZ exhibited a distinct profile with upregulation of xenobiotic metabolism and reactive oxygen species pathways, suggesting oxidative stress as a central mechanism, along with altered cholesterol and androgen responses. Furthermore, Gene Ontology (GO) analysis confirms the same molecular convergence as observed with the MsigDb hallmark analysis. These findings suggest shared inflammatory and signaling pathway disruptions across these psychiatric conditions, while also revealing disorder-specific molecular signatures that could inform targeted therapeutic approaches.

These molecular signatures correlate remarkably with clinical presentations: the dysregulated stress response and metabolic pathways in MD align with observed neurovegetative symptoms and stress sensitivity (22); the oscillating cellular signaling patterns in BD mirror the cyclic nature of mood states (23); and the combination of immune activation and oxidative stress in Schizophrenia may underlie the progressive nature of cognitive symptoms (24). Of therapeutic relevance, these findings suggest that while immune-modulating strategies might benefit BD and SCZ patients, alternative approaches targeting metabolic and stress response pathways might be more effective for MDD. Furthermore, the identification of disorder-specific pathway dysregulation provides potential novel therapeutic targets: mTOR pathway modulators for BD, antioxidant strategies for SCZ, and metabolic pathway interventions for MDD.

## Immune cell composition analysis across psychological disorder

**Figure 5:**
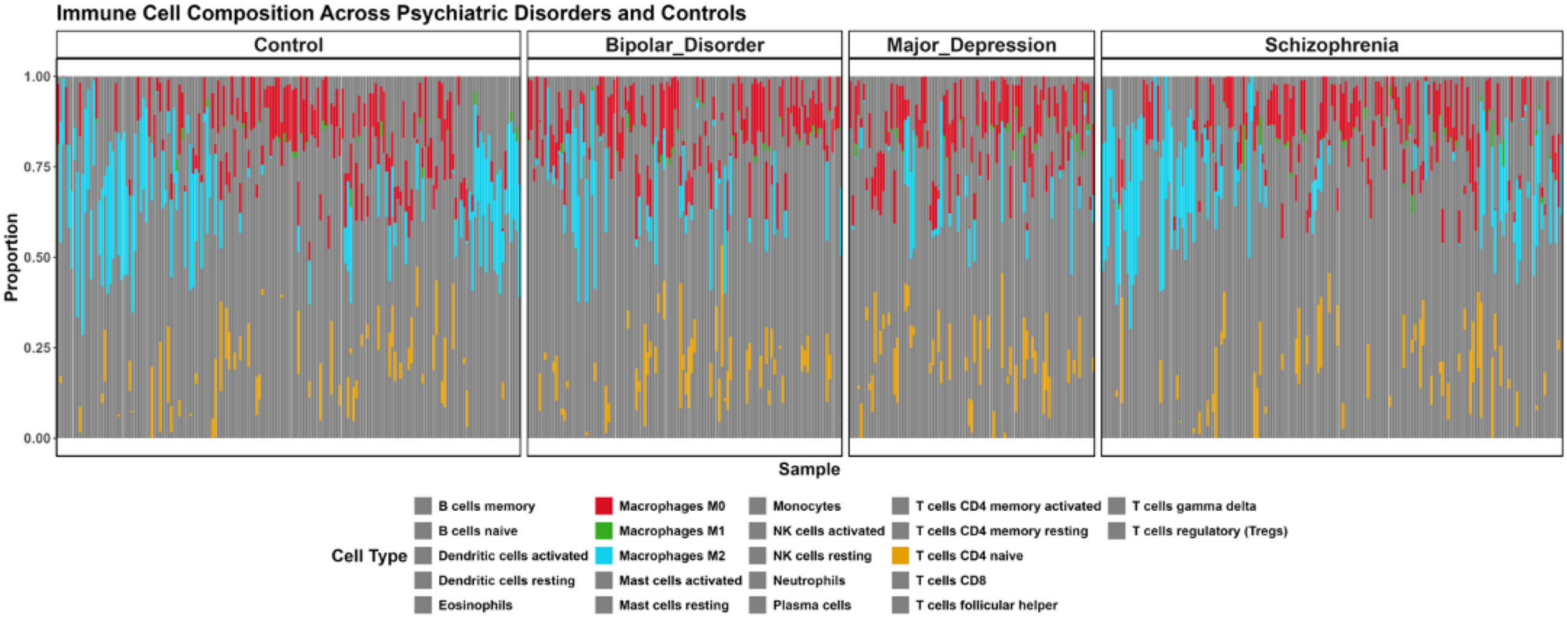
Immune Cell Composition Across Major Psychiatric Disorders Based on CIBERSORT Deconvolution. Brain tissue samples from controls and patients with Bipolar Disorder, Major Depression, and Schizophrenia analyzed for 22 immune cell subtypes using LM22 signature matrix (1000 permutations, p < 0.05). Each vertical bar represents an individual sample with colored segments showing relative proportions (0-1.0 scale) of immune cell populations. Only cell types which shows significant difference in proportion compared to the control (pval < 0.05) shown in bright color, the cell type which are not significant are shown in grey color.

Since pathway analysis results in varied expression of immune signature across the disorder. In order to better deconvolute the immune cell governing each disorder, we sought to perform the immune cell deconvolution using CIBERSORT algorithm (25) with LM22 gene signature. CIBERSORT immune cell deconvolution reveals distinct immune cell compositional differences between BD, MDD, and Schizophrenia. Macrophages M0 exhibited significantly higher proportions in Bipolar Disorder compared to Controls (p=0.008408421), in Major Depression compared to Controls (p=0.007015745), and were also significantly different when comparing Schizophrenia to both Bipolar Disorder (p=0.007015745) and Major Depression (p=0.007015745). This suggests an overall elevated presence of M0 macrophages in these disorders, particularly pronounced in Schizophrenia. Macrophages M1 showed a significantly higher proportion in Major Depression compared to Controls (p=0.023905212). Furthermore, Macrophages M2 demonstrated significantly altered proportions in Bipolar Disorder compared to Controls (p=0.005258366), in Major Depression compared to Controls (p=001229292), and were significantly different when comparing Schizophrenia to Major Depression (p=0.040085284). Regarding T cell populations, T cells CD4 naive showed significantly reduced proportions in Major Depression compared to Controls (p=0.039030542), and were also significantly lower when comparing Schizophrenia to Major Depression (p=0.039030542). (**Figure 5**; **Supplementary Table S4**; **Supplementary Figure 2**). The consistent presence but varying proportions of Macrophages (M0, M1, M2) across disorders indicates potential differential polarization of myeloid cells that may contribute to disorder-specific inflammatory environments. These findings align with emerging evidence of immune dysregulation in psychiatric disorders.

## Transcription factor enrichment analysis uncovers distinctive regulatory programs

To investigate the observed disease-specific gene expression and pathway alterations, we performed transcription factor (TF) enrichment analysis to prioritize regulatory elements governing these behaviors. Using ChIP-X Enrichment Analysis 3 (ChEA3) on leading-edge genes from MSigDB pathway analysis, we identified unique TF regulatory signatures across all three psychiatric conditions. This approach systematically evaluates which transcription factors are most likely to regulate the disease specific behavior comparing them against curated libraries of known TF-target relationships derived from diverse genomic datasets. The ChEA3 employs Fisher’s exact test with a reference background of 20,000 genes to statistically assess the overlap between our disease-associated gene signatures and established TF regulatory networks, enabling identification of transcriptional drivers that may orchestrate the observed pathological gene expression programs in each psychiatric condition.

**Figure 6:**
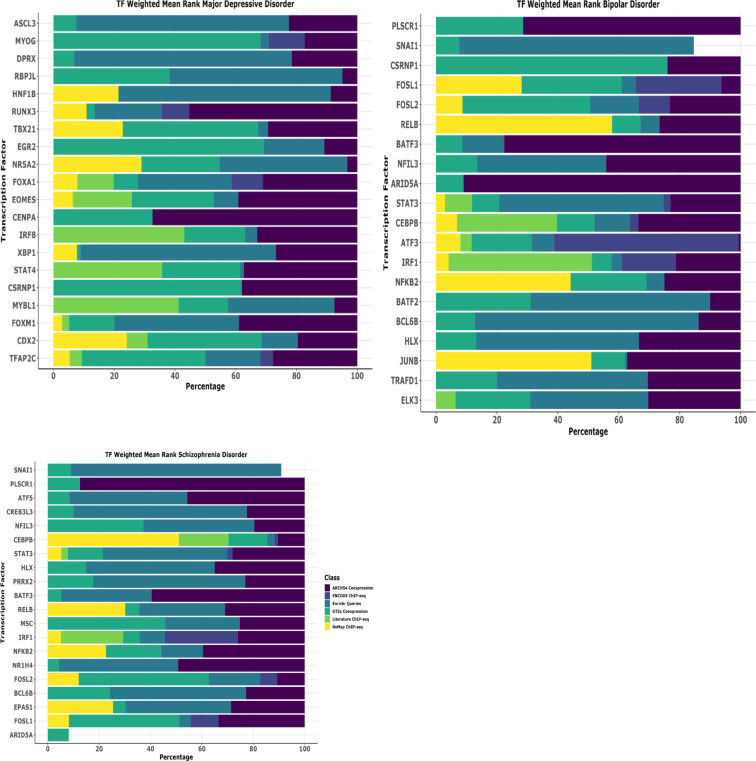
Transcription factor uncovers the unique biology of disease. Comprehensive transcription factor (TF) enrichment analysis identifying regulatory drivers of disease-specific gene expression programs in schizophrenia disorder (SCZ). Analysis performed using ChIP-X Enrichment Analysis 3 (ChEA3) database on leading-edge genes derived from MSigDB pathway enrichment analysis. ChEA3 integrates multiple evidence sources including: (1) ARCHS4 Coexpression data (purple), (2) ENCODE ChIP-seq binding data (dark blue), (3) Enrichr Queries database (teal), (4) GTEx Coexpression data (green), (5) Literature ChIP-seq studies (light green), and (6) ReMap ChIP-seq database (yellow), generating composite ranking scores for statistical significance. The horizontal stacked bar chart displays the top 20 transcription factors for each disease category, ranked by weighted mean rank percentages across all evidence classes. Each colored segment represents the contribution of different evidence sources to the overall TF ranking, with the total bar length indicating the combined weighted mean rank percentage. TFs are ordered by overall composite ranking significance.

Our comprehensive analysis revealed the top 20 transcription factors for each condition based on composite ranking scores that integrate multiple evidence sources, with notable disease-specific patterns. For major depressive disorder (MDD), the most significantly enriched TFs were ASCL3, MYOG, DPRX, RBPJL, HNF1B, RUNX3, TBX21, EGR2, NR5A2, FOXA1, EOMES, CENPA, IRF8, XBP1, STAT4, CSRNP1, MYBL1, FOXM1, CDX2, TFAP2C representing the highestranking factors with strongest statistical support. These top-ranked TFs primarily function in neurodevelopmental processes, neuronal differentiation, regulating muscle development and neuroplasticity, controlling developmental gene programs as shown by literature evidence. In bipolar disorder (BD), the transcription factor landscape was dominated by immune and inflammatory regulatory elements. The top-ranked TFs were PLSCR1, SNAI1, CSRNP1, FOSL1, FOSL2, RELB, and BATF3, NFIL3, ARID5A, STAT3, CEBPB, ATF3, IRF1, NFKB2, BATF2, BCL6B, HLX, JUNB, TRAFD1, and ELK3 which collectively orchestrate immune-related gene expression changes. Schizophrenia (SCZ) exhibited a unique regulatory profile characterized by transcription factors involved in both immune function and neurodevelopmental processes. The most significantly enriched factors were SNAI1, PLSCR1, ATF5, CREB3L3, NFIL3, CEBPB, and STAT3, HLX, PRRX2, BATF3, RELB, MSC, IRF1, NFKB2, NR1H4, FOSL2, BCL6B, EPAS1, FOSL1, ARID5A (**Figure 6**). This collectively demonstrate the dual immune-neurodevelopmental signature, suggesting convergent dysregulation of these pathways in SCZ etiology.

## Literature validation of the key genes and identification of drug targets

To further explore the key biological difference observed at the disease level, we sought to validate our findings with literature search. Using the DGE list, we searched the genes across the web for their involvement in pathophysiology across the disease status. In BD, several of our identified genes such as SERPINA3 (26), CCL2 (27), SOCS3 (27), S100A3 (28), FOSL1 (29) (**Figure 4**, volcano plot) have been previously implicated in pathophysiology observed bipolar patients. Notably, in SCZ, genes such as SERPINA3 (30), CHI3L1 (31), SOCS3 (32), CASP1 (32), IL1RL1 (33), IL6 (33), HBG2 (34), GRIN2A (35) and GRIA3 (36) have established associations with inflammation, synaptic plasticity and disease severity. For MDD, our key genes, including CHI3L1 (37), SERPINA3 (38), CP, which involved in metabolism (https://psychiatrypsychopharmacology.com/en/ceruloplasmin-levels-before-and-after-treatment-in-patients-withdepression-a-case-control-study-132758) align with published studies demonstrating their roles in stress and mood regulation. Next, to explore the therapeutic potential of our DGE gene list in a disease-specific manner, we utilized the DGIdb resource (https://www.dgidb.org/search_interactions), which catalogs the experimentally validated druggene interaction. Our drug-gene interaction analysis using DGIdb uncovered several promising therapeutic implications across psychiatric disorders. Notably, SERPINA3, a key dysregulated gene in our analysis, showed interactions with multiple established antipsychotic agents including risperidone, olanzapine, and clozapine, validating its relevance in psychiatric pathophysiology. The inflammatory mediators identified in our study, particularly IL6 and CCL2, demonstrated interactions with various therapeutic agents, including immunomodulators and antipsychotics, CP with antidepressants etc. The DGIdb analysis uncovered several promising drug-gene interactions beyond our curated psychiatric gene set. For example - SLC22A12 demonstrated interactions with multiple therapeutic agents, including losartan and antineoplastics, suggesting potential metabolic pathway interventions. PTGIR showed significant associations with cardiovascular agents like selexipag and epoprostenol, highlighting possible vascular-related therapeutic approaches. The CALCA pathway revealed interactions with novel therapeutic antibodies (galcanezumab, fremanezumab) and traditional medications, suggesting its potential role in pain and neurotransmitter modulation. CHRNG’s interactions with multiple neuromuscular blocking agents point to possible therapeutic implications for motor symptoms. PLA2G2A’s connections to antiinflammatory agents and corticosteroids, along with CXCR1/2’s interaction profile with antiinflammatory compounds, suggest additional inflammatory pathway intervention possibilities. Notably, NPC1L1’s interaction with lipid-modulating drugs like ezetimibe indicates potential metabolic therapeutic approaches. These previously unexplored drug-gene interactions reveal additional therapeutic opportunities and potential drug repurposing strategies for psychiatric disorders, particularly through modulation of inflammatory, metabolic, and neurotransmitter pathways. The summarized list of drug-gene interaction has been added into the **Supplementary Table S5**.

## Key highlights of the present study

The present study comprehensive transcriptomic analysis of 557 RNA-seq datasets across Schizophrenia (SCZ), Bipolar Disorder (BD), and Major Depressive Disorder (MDD) provides novel insights into psychiatric disorder molecular mechanisms. The study reveals: (1) significant molecular convergence between SCZ and BD with 373 shared differentially expressed genes, substantiating their clinical similarities; (2) distinct disorder-specific transcriptional profiles, particularly BD’s complex molecular landscape; (3) brain region-specific molecular alterations, especially in hippocampus and dorsolateral prefrontal cortex, highlighting spatial heterogeneity in psychiatric disorders; (4) disorder-specific pathway disruptions including stress response and metabolic dysregulation in MDD, immune activation in BD, and immune-oxidative stress interactions in SCZ; (5) identification of key transcription factors as major regulators of each psychiatric disorder; and (6) promising therapeutic strategies through drug-gene interaction analysis, including SERPINA3’s interaction with antipsychotics and IL6/CCL2 with immunomodulators. These findings advance precision psychiatry by elucidating the molecular complexity underlying these disorders and offer a foundation for developing targeted treatment approaches. In conclusion, the present study offers a multi-disorder, multi-region transcriptomic perspective on psychiatric disorders, identifying key molecular pathways, transcription factors, and drug-gene interactions that could inform precision medicine strategies.

## Discussion

Our comprehensive transcriptomic analysis reveals both shared and unique molecular signatures across SCZ, BD, and MDD, providing crucial insights into the biological foundations of these psychiatric conditions. The identification of 373 common differentially expressed genes (DEGs) between SCZ and BD and 12 common hub genes across all three disorders, supports the hypothesis of shared pathophysiological mechanisms, while distinct transcriptional patterns highlight disorder-specific molecular pathways. This substantial molecular overlap between BD and SCZ provides a molecular basis for the clinical similarities often observed between these disorders and may explain the challenges clinicians face in differential diagnosis.

The observation that BD exhibited the largest number of unique DEGs suggests a particularly complex molecular landscape, potentially reflecting the disorder’s characteristic oscillation between manic and depressive states. This finding aligns with the robust immune system activation and cellular growth signaling perturbations observed in BD patients, suggesting potential therapeutic targets specific to this condition. Brain region-specific transcriptional patterns, particularly in the hippocampus and DLPFC, underscore the spatial heterogeneity of gene expression changes in psychiatric disorders. These findings suggest that therapeutic approaches may need to consider both disorder-specific and region-specific molecular alterations. Our analysis of pathway disruption reveals complex interactions between different biological systems, particularly notable in the immune system’s involvement across all three disorders, albeit with varying patterns and intensity. The distinct pathway dysregulation patterns observed for each disorder – stress response and metabolic pathways in MDD, immune system activation in BD, and a combination of immune dysregulation and oxidative stress in SCZ – provide potential targets for tailored therapeutic interventions. This finding suggests that immune modulation might represent a promising therapeutic avenue, though the approach would need to be carefully tailored to each disorder’s specific immune signature. The disorder-specific molecular signatures could guide the development of novel therapeutic agents, potentially leading to more precise treatment strategies that address the unique pathophysiological mechanisms of each condition.

Several limitations of this study warrant consideration. First, the use of publicly available datasets introduces potential heterogeneity in sample collection and processing methods. Second, transcriptomic analysis of post-mortem tissue provides only a terminal snapshot of gene expression, potentially missing the dynamic molecular changes that occur throughout disease progression. Future longitudinal studies could help address this limitation. The lack of available data regarding the medication history of the subjects in the datasets used is further limitation of the study, as the potential effects of psychiatric medications on gene expression could not be accounted in the analysis. Additionally, the focus on specific brain regions, while providing detailed insights, may not capture the full complexity of brain-wide network disruptions in psychiatric disorders.

Future research directions should address these limitations through multiple approaches. Validation of key molecular signatures could be pursued through complementary methods such as single-cell RNA sequencing of post-mortem tissue (39, 40), which might better account for cellular heterogeneity and provide higher resolution of cell-type-specific changes. The development of improved methods for handling post-mortem tissue and standardizing collection procedures across brain banks would enhance data quality and reproducibility (41, 42). Integration of these findings with other molecular data types especially proteomic (43), metabolomic (44), and epigenetic data (44) could provide a more comprehensive understanding of the biological mechanisms underlying psychiatric disorders. Additionally, future studies should consider alternative approaches such as patient-derived induced pluripotent stem cells (iPSCs) (45, 46) and brain organoids (47), which could help overcome some limitations of post-mortem studies by enabling longitudinal analysis and investigation of developmental aspects of these disorders. These models, while having their own limitations, could complement post-mortem studies and provide insights into the temporal dynamics of disease progression.

In conclusion, our study provides valuable insights into the molecular landscape of major psychiatric disorders, revealing a complex interplay of shared biological mechanisms and disorderspecific pathways. Our findings reinforce the idea of a biological continuum across major psychiatric disorders, with shared molecular alterations suggesting overlapping pathophysiological mechanisms despite clinical differences. The identification of distinct transcriptional signatures and key regulatory networks contributes significantly to our understanding of the biological continuum across psychiatric conditions. These findings not only suggest potential targets for therapeutic intervention but also emphasize the importance of considering both common and unique molecular features in treatment development. These findings offer valuable guidance for the development of more personalized treatment strategies in psychiatry by identifying potential therapeutic targets and drug repurposing opportunities. The study also underscores the importance of tailoring interventions based on disorder-specific and brain region-specific molecular profiles. As we move forward, validating these transcriptomic signatures in independent and longitudinal cohorts will be essential to strengthen their clinical relevance. Integrating these molecular insights with patient phenotypes and treatment responses could pave the way for biomarker discovery and more personalized therapeutic strategies. Furthermore, the incorporation of multi-omics data and functional studies, such as gene editing or single-cell analyses, may refine our understanding of disease mechanisms and advance precision medicine approaches in psychiatric care.

## Data Availability

The original contributions presented in the study are included in the article/**Supplementary Material**. Further inquiries can be directed to the corresponding authors.
