# Supplementary figures and images for: Deciphering transcriptomic signatures in schizophrenia, bipolar disorder, and major depressive disorder
Source: Front Psychiatry. 2025 Jul 14;16:1574458. doi: 10.3389/fpsyt.2025.1574458 (PMC12301984; doi:10.3389/fpsyt.2025.1574458)

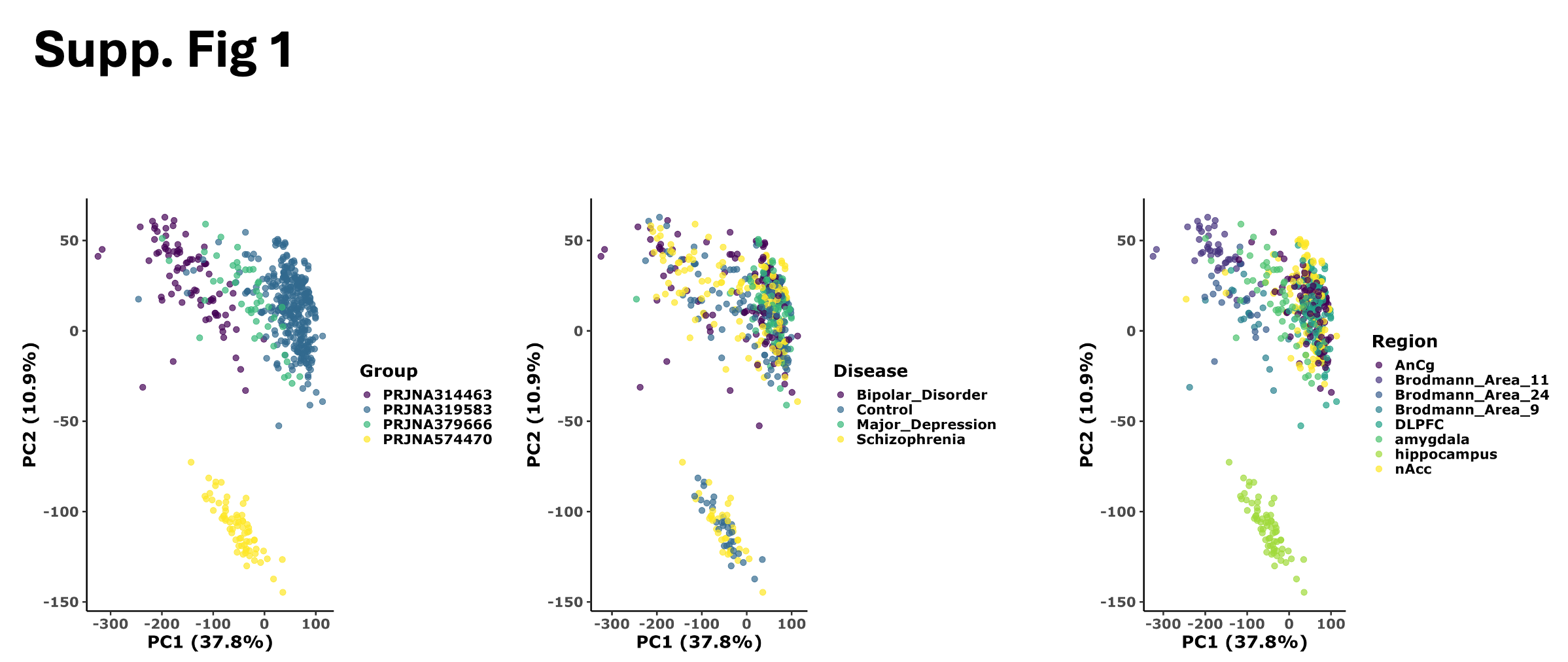

Supplement: Supplementary file 1 [file Image1.png]

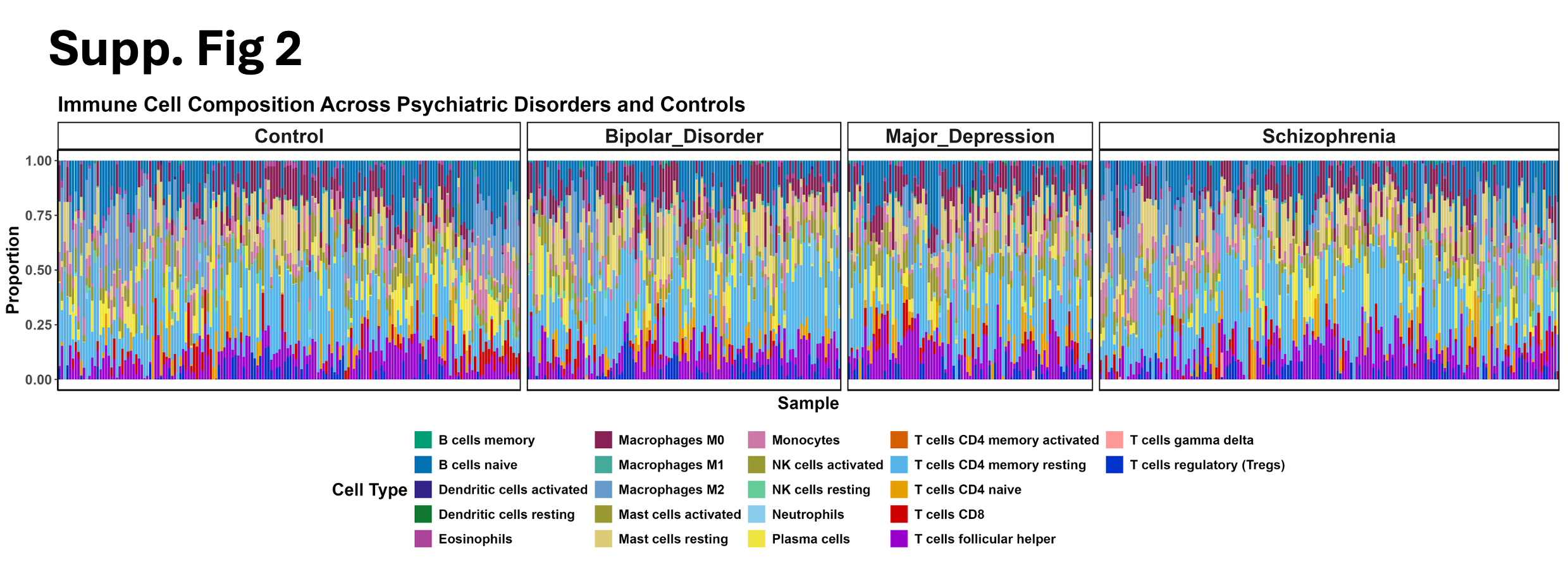

Supplement: Supplementary file 2 [file Image2.png]
